# IL-17 in the Rheumatologist's Line of Sight

**DOI:** 10.1155/2013/295132

**Published:** 2013-07-25

**Authors:** Marie-Elise Truchetet, M. Djavad Mossalayi, Katia Boniface

**Affiliations:** ^1^UMR-CNRS 5164, Composantes Innées de la Réponse Immunitaire et de la Différenciation, Université Bordeaux Segalen, 146 rue Léo Saignat, 33076 Bordeaux, France; ^2^Département de Rhumatologie, Hôpital Pellegrin, CHU de Bordeaux, place Amélie-Raba-Léon, 33076 Bordeaux, France; ^3^INSERM U1035–Biothérapies des maladies génétiques et cancers, Université Bordeaux Segalen, 146 rue Léo Saignat, 33076 Bordeaux, France

## Abstract

Over the past decades, the identification of several new cytokines, including interleukin (IL)-17 and IL-23, and of new T helper cell subsets, including Th17 cells, has changed the vision of immunological processes. The IL-17/Th17 pathway plays a critical role during the development of inflammation and autoimmunity, and targeting this pathway has become an attractive strategy for a number of diseases. This review aims to describe the effects of IL-17 in the joint and its roles in the development of autoimmune and inflammatory arthritis. Furthermore, biotherapies targeting directly or indirectly IL-17 in inflammatory rheumatisms will be developed.

## 1. Introduction

Cytokines play a key role in the coordination of the innate and adaptive immune responses to protect an organism against internal and external pathogenic assault. Over the past decades, the identification of several new cytokines, including interleukin (IL)-17 (also known as IL-17A) and IL-23, has changed the vision of immunological processes.

In response to antigen stimulation, naive CD4^+^ T cells differentiate into different T cell subsets with specialized effector functions, mainly on the basis of their cytokine expression profile. T helper type 1 (Th1) cells develop in response to IL-12 and produce high amounts of interferon (IFN)-*γ*, required to control infection with intracellular pathogens such as viruses. This cell subset is also important during inflammation and autoimmunity. IL-4 is the major inducer of Th2 cells that produce IL-4, IL-5, and IL-13, which are crucial for the clearance of parasitic worms and during development of allergic inflammation. The Th1/Th2 dichotomy paradigm has been revisited with the recent identification of additional effector CD4^+^ T cell subsets producing IL-17 (Th17), IL-22 (Th22), or IL-9 (Th9) [1–6]. The importance of Th17 cells during development of autoimmune and inflammatory diseases is now well documented. These cells play also a critical role during defense against extracellular pathogens. Besides Th17 cells, *γδ* T cells, innate lymphoid cells, natural killer cells, and CD8^+^ T cells represent other and important sources of IL-17.

This review aims to overview the role of IL-17 during host defense and autoimmunity, with a particular focus on IL-17 and articular inflammation. Biotherapies targeting directly or indirectly this cytokine in inflammatory rheumatisms will also be developed.

## 2. IL-17: Signaling, Cellular Sources, and Biological Activities

### 2.1. IL-17 and IL-17 Receptor Signaling

**Figure 1:**
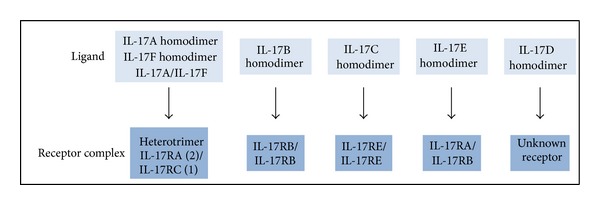
IL-17 cytokines and receptors family.

Originally called cytotoxic T-lymphocyte-associated antigen 8 (CTLA8), IL-17 was first identified in rodent T cell hybridoma clones and subsequently cloned from human CD4^+^ T cell library [7–9]. It is the founding member of the IL-17 cytokine family, which is composed of six members: IL-17 (IL-17A), IL-17B, IL-17C, IL-17D, IL-17E (IL-25), and IL-17F. IL-17 and IL-17F are highly homologous and bind the same receptor, implying shared biological activities (Figure 1). In addition, IL-17 exists as a homodimer or as a heterodimer with IL-17F [10, 11].

The IL-17 receptor family contains five members, from IL-17RA to IL-17RE, and functional receptors for IL-17 cytokine family consist of homo- or heterodimers (Figure 1). Both IL-17 and IL-17 receptor family members have little homology to other known cytokines and cytokine receptors and are thus classified as a new cytokine and cytokine receptor families.

IL-17 acts through a heterotrimeric receptor composed of two IL-17RA chains and one IL-17RC subunit [11, 12]. Such receptor complex is shared with IL-17F and IL-17A/IL-17F heterodimer. IL-17RA is ubiquitously expressed, with elevated levels in hematopoietic cells; however, IL-17 main responsive cells are epithelial and endothelial cells, fibroblasts, and to a lesser extent macrophages, dendritic cells, and B cells. In contrast, IL-17RC is weakly expressed in hematopoietic cells, and higher expression is observed in nonhematopoietic tissues, such as liver, prostate, and joints. Thus, IL-17RA and IL-17RC differential expression may explain tissue-specific function of IL-17. Binding of IL-17 to IL-17RA induces recruitment of IL-17RC to form an active IL-17RA/IL-17RC complex, inducing mitogen-activated protein (MAP) kinases, nuclear factor *κ* B (NF*κ*B), phosphoinositide 3 kinase (PI3K), and C/EBP signaling pathways [11]. In addition, NF*κ*B activator 1 (Act 1), an adaptor protein for IL-17 receptor, is an essential component of IL-17-mediated signaling and downstream effects [13, 14].

As detailed below, IL-17 is mainly known for its roles in host defense, inflammation, and autoimmunity, and its expression is increased in inflammatory tissues [15].

### 2.2. IL-17: Adaptive and Innate Sources

Whereas IL-17 levels are low or undetected in normal homeostatic conditions, IL-17 production is highly increased following diverse stimuli, including infection and inflammation. Elevated IL-17 expression is also observed in a number of autoimmune and inflammatory diseases. IL-17-producing cells mainly belong to the hematopoietic lineage, comprising both innate and adaptive immune cells. Interestingly, both IL-1*β* and IL-23 are potent inducers of IL-17 production by these cell subsets.

#### 2.2.1. Adaptive Sources of IL-17

IL-17 has been known to be produced by T cells for the past 18 years; however, the identification of IL-17-producing CD4^+^ T (Th17) cells as a T helper cell subset distinct from Th1 and Th2 cells [1–3] has had a tremendous impact on our understanding of the cytokines and T cell pathways that are involved during development and maintenance of chronic inflammation. Th17 cells were first recognized when assessing the role of IL-23 in various mouse models of chronic inflammation and autoimmunity, including inflammatory bowel diseases (IBDs), collagen-induced arthritis (CIA), or experimental autoimmune encephalomyelitis (EAE, a murine model of multiple sclerosis) [2, 16, 17]. In addition to IL-23, IL-1*β*, IL-21, prostaglandin E2 (PGE2), transforming growth factor (TGF)-*β*, and IL-6 regulate development of this cell subset [16–25]. Furthermore, retinoic acid receptor-related orphan receptor-*γ*t (ROR*γ*t), ROR*α*, signal transducer and activator of transcription 3 (STAT3), Interferon regulatory factor 4 (IRF4), and aryl hydrocarbon receptor (AHR) are key transcription factors in the differentiation program of Th17 cells [26–30]. Mammalian target of rapamycin (mTOR) and hypoxia-inducible factor 1*α* (HIF1*α*) were also recently identified as factors positively regulating Th17 development [31–34]. Although Th17 cells derived their name because of their ability to secrete IL-17, they also produce elevated levels of IL-17F, IL-22, IFN-*γ*, tumor necrosis factor (TNF)-*α*, IL-6, and CCL20, which have both overlapping and distinct roles during inflammation and host defense [16, 35]. Th17 cells have been largely described for their key role in the pathogenesis of inflammatory and autoimmune disorders, including arthritis, IBD, psoriasis, and multiple sclerosis, and targeting the Th17 pathway is showing promising results for treatment of chronic inflammation [36].

Although IL-17 is considered a CD4^+^ T cell product, activated CD8^+^ T cells are another adaptive source of this cytokine [37–39]. In line with distinct subsets of CD4^+^ T cells, naive CD8^+^ T cells can be polarized into different effector phenotypes, such as type 1 (Tc1), type 2 (Tc2) cells, and the recently described IL-17-producing CD8^+^ T cell subset, defined as Tc17 [40–43]. Tc17 cells display reduced cytotoxic activity and express molecules of the Th17 program. Data from an increasing number of reports suggest a possible role of Tc17 cells during inflammation and autoimmunity [44–47].

Lastly, B cells were very recently identified as an important source of IL-17 in response to *Trypanosoma cruzi* infection both in mice and human [48]. Such IL-17 production is independent of ROR*γ*t, ROR*α*, and AHR and is unaffected after *T. cruzi* in IL-6 or IL-23 receptor deficient mice, showing that, in contrast to other cellular sources of IL-17, B cells do not use the canonical IL-17 program.

#### 2.2.2. Innate Sources of IL-17

IL-17 production by adaptive immune cells could not explain the existence of early IL-17-mediated immune responses, and a wide range of studies have shown that IL-17 is also produced by a variety of innate cell subsets, including *γδ* T cells, innate lymphoid cells, and natural killer cells [49, 50]. Whether mast cells and neutrophils can produce IL-17 is still under investigation. IL-1*β*, IL-23, and downstream-activated transcription factors, ROR*γ*t, STAT3, and AHR, have been described as important factors to induce innate IL-17-producing cell development [49, 51]. These innate sources of IL-17 play a crucial role during stress responses and mucosal host defense. In addition, innate IL-17 producers have been involved in the development of autoimmune diseases, such as EAE, arthritis, and colitis [50–54].

### 2.3. IL-17 in Host Defense and Autoimmunity

Since its identification, biological activities of IL-17 have been extensively investigated. This cytokine has pleiotropic effects that bridge innate and adaptive immunity and plays critical roles during host defense against pathogens, as well as during development and maintenance of autoimmune and inflammatory diseases.

IL-17 promotes expression of antimicrobial peptides by keratinocytes, lung, and gut epithelial cells, such as defensins, S100A proteins, and lipocalin 2. It also induces secretion of proinflammatory cytokines (e.g., IL-1, IL-6, and TNF-*α*), chemokines (e.g., IL-8, CCL20, CCL2, and CXCL5), and matrix metalloproteinases (e.g., MMP1, MMP3, and MMP9) from multiple target cells, including epithelial and endothelial cells, fibroblasts, neutrophils, and osteoblasts [55, 56]. Such effects explain the diversity of IL-17 biological activities in the organism: promotion of inflammation, protection against infection, and chemotactic effects that induce recruitment of Th17 cells, as well as innate cells, such as neutrophils. Interestingly, IL-17 also cooperates with other cytokines to promote inflammation, such as TNF-*α*, IL-6, and IL-1*β*.

#### 2.3.1. IL-17 in Host Defense

IL-17, as well as Th17-related cytokines IL-17F and IL-22, protects hosts against several microbial and fungal pathogens at epithelial and mucosal tissues, including skin, intestine, and lung. IL-17-signaling deficiency in mice causes a dramatic reduction in neutrophil chemotaxis and a subsequent increased susceptibility to bacterial infection. For example, mice deficient in IL-17 and/or IL-17RA show increased susceptibility to infections with *Klebsiella pneumonia*, *Staphylococcus aureus*, *Citrobacter rodentium*, and *Candida Albicans* [57–61]. In addition, emerging evidence points to an involvement of IL-17 and Th17 cells during immune protection against parasites and viruses [61–64]. Interestingly, Bermejo et al. just identified IL-17-producing B cells as critical to control trypanosome infection [48].

Several reports support an important role of Th17 cells during host defense in humans. The IL-23/Th17 pathway is important during defense against *Mycoplasma hominis* [65]; human memory T cells specific for *C. albicans* belong to the Th17 lineage [66], and patients with chronic mucocutaneous candidiasis, a heterogeneous group of disorders characterized by recurrent or persistent infections (predominantly with *C. albicans* and to a lesser extent with *S. aureus*), have reduced production of IL-17 and IL-22 [67].

Recent genetic studies revealed disease susceptibility association with IL-17RA autosomal recessive deficiency [68]. Lastly, patients suffering from hyper-IgE syndrome are highly susceptible to bacterial and fungal infections and have impaired Th17 cell differentiation [69–71]. However, the exact contribution of Th17 cells versus innate immune cells for protective immunity still needs to be fully determined.

#### 2.3.2. IL-17 in Autoimmunity

In contrast to their protective role during host defense, IL-17 and other Th17-related cytokines (i.e., IL-22, IL-17F) can have adverse effects resulting in tissue damage. Th17 cells are linked to the pathogenesis of various human autoimmune and inflammatory diseases, and IL-17, IL-17F, IL-22, and IL-23 levels are increased in RA, psoriasis, multiple sclerosis, and IBD [4, 17, 72–75]. Together with Th17 cells, mast cells, neutrophils, Tc17, and *γδ*-T cells represent additional sources of IL-17 in inflammatory diseases [76–78]. IL-17-producing *γδ*-T cells are involved in the development of skin, brain, and articular inflammation *in vivo* [52, 79–81]. In addition, Tc17 cells cooperate with Th17 cells for the induction of EAE [47].

Consistent with these observations, studies in mice deficient in IL-17 or its receptor and blockade of IL-17 or IL-17 receptor revealed an important role of IL-17 *in vivo* during induction and propagation of autoimmunity in animal models, such as EAE and CIA [82–86]. Interestingly, IL-23 appears as an essential cytokine to drive the pathogenicity of both innate and adaptive IL-17-producing cells [87, 88].

### 2.4. IL-17 in the Joint

**Figure 2:**
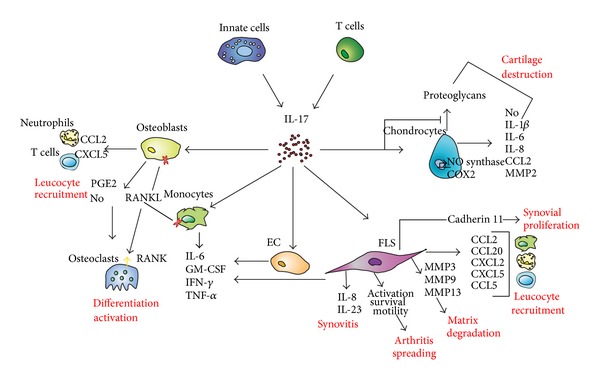
Effects of IL-17 in the joint. COX2: cyclooxygenase 2; EC: endothelial cells; FLS: fibroblast-like synoviocytes; GM-CSF: granulocyte-monocyte colony-stimulating factor; IFN-*γ*: interferon-*γ*; IL: interleukin; MMP: matrix metalloproteinases; NO: nitric oxide; PGE2: prostaglandin E2; RANK: receptor activator of NF*κ*B; RANKL: RANK ligand; TNF-*α*: tumor necrosis factor-*α*.

Together with IL-1*β*, TNF-*α*, and IL-23, IL-17 is an additional cytokine able to promote articular inflammation and damage (Figure 2). As detailed in the next part of this review, elevated levels of IL-17 are found in patients with autoimmune or inflammatory rheumatisms, such as RA, spondyloarthritis (SpA), systemic lupus erythematosus (SLE), or systemic sclerosis (SSc) [89–93], and *in vivo* studies demonstrated an important role of IL-17 in autoimmune arthritis by aggravating synovial inflammation and joint destruction [94, 95]. Conversely, IL-17 deficiency or inhibition protects from joint inflammation and damage in animal models of arthritis [82, 96, 97]. Besides Th17 cells, innate immune cells are also an important source of IL-17 in inflammatory joint diseases, and both Th17 cells and innate IL-17 producers have been shown to be important players of IL-17-induced effects in the joint [52, 98–104].

#### 2.4.1. IL-17 and Bone Metabolism

IL-17 affects bone remodeling through its effects on osteoblasts and osteoclasts; it induces production of PGE2, nitric oxide (NO), and receptor activator of NF*κ*B ligand (RANKL) by osteoblasts, leading to osteoclast differentiation and activation, indirectly in favor of bone destruction. Interestingly, it was recently shown that IL-17 can also directly induce osteoclastogenesis from human monocytes in the absence of osteoblasts, and such effect is TNF-*α* dependent [105]. IL-17 also upregulates production of proinflammatory cytokines, such as IL-6, granulocyte-monocyte colony-stimulating factor (GM-CSF), IFN-*γ*, and TNF-*α* by fibroblasts, epithelial, endothelial cells, monocytes, and bone cells, also in favor of bone loss [89, 106–109]. In addition, IL-17 increases expression of chemotactic factors by osteoblasts, such as CCL2 and CXCL5, promoting recruitment of leucocytes, including neutrophils and T cells [108, 110], that are able to produce factors (e.g., IL-6, IL-1, TNF-*α*, and RANKL) that will further affect bone resorption.

#### 2.4.2. IL-17 and Synovial Inflammation

IL-17 promotes joint degradation by acting on synoviocyte activation, survival, and migration. It increases expression of inflammatory cytokines and chemokines by synoviocytes, such as IL-6, IL-8, CCL20, TNF-*α*, and IL-23p19 subunit. IL-17 also contributes to the production of matrix metalloproteinases by the cells, including MMP3, MMP9, and MMP13, which drive degradation of the extracellular matrix within the joint [111–116]. Furthermore, IL-17 can synergize with other inflammatory cytokines, such as TNF-*α*, IL-1*β*, or IL-17F in synoviocytes [114, 117]. IL-17-induced chemokine production (e.g., IL-8, CXCL2, CCL20, CCL2, CXC5, and CCL5) by various cell types, including synoviocytes and synovial macrophages, contributes to recruitment of neutrophils, lymphocytes, and macrophages to the synovium, thereby enhancing inflammation [3, 108, 113, 118, 119]. Interestingly, IL-17 increases cadherin-11 expression in patients with RA as well as in mice with CIA, an adhesion molecule contributing to synovial inflammation and cartilage degradation [120]. IL-17 also increases IL-6 production by RA synovium explants while inhibiting type 1 collagen synthesis [121]. Recent data reported by Kato et al. suggest that IL-17 produced by Th17 cells is more important in the induction of proinflammatory cytokines rather than in the induction of cell-cell interaction molecules by synoviocytes, two relevant components of synovial inflammation [122]. In addition, IL-17 increases survival and motility of synoviocytes from RA patients [117, 123], and it was reported that migration of activated RA synoviocytes has the ability to spread arthritis to unaffected joints [124].

Formation of new vessels largely contributes to the formation and maintenance of the pannus in RA and, therefore, to cartilage and bone damage. IL-17 also contributes to angiogenesis by increasing production of proangiogenic factors by synoviocytes, such as vascular endothelial growth factor [125, 126].

#### 2.4.3. IL-17 and Cartilage Remodeling

Another important target in joint inflammation is cartilage, and destruction of cartilage is a major consequence of chronic synovitis. Stimulation of normal and osteoarthritic human chondrocytes with IL-17 induces NO production as well as expression of genes and proteins associated with joint inflammation and cartilage degradation, such as inducible NO synthase, cyclooxygenase 2, IL-1*β*, IL-6, IL-8, CCL2, and MMP. These effects are mediated through activation of the MAP kinases, NF*κ*B, and AP-1 signaling pathways [127–129]. In line with these observations, IL-17 inhibits proteoglycan synthesis by cartilage, and intra-articular administration of IL-17 in mice leads to cartilage destruction [121, 130]. Effects of IL-17 and IL-17 receptor signaling in promoting cartilage damage have been further confirmed *in vivo* in mouse models of CIA, and blocking IL-17 or IL-17 receptor deficiency reduces cartilage degradation [94, 97, 111]. Furthermore, IL-17 can synergize with other proinflammatory cytokines, such as TNF-*α*, to promote cartilage destruction [128, 131].

#### 2.4.4. IL-17 and Enthesitis

The enthesis, located at the junction of tendon to bone, is the primary site of articular inflammation in SpA. Increased levels of IL-23 and IL-17 have been observed in sera from patients with SpA, like ankylosing spondylitis (AS) [90, 132–134]. Importantly, IL-23 was very recently identified as a major cytokine driving entheseal inflammation *in vivo* [135]. Notably, IL-23-sensitive cells in entheses are resident CD3^+^CD4^−^CD8^−^ROR*γ*t^+^ T cells. They allow the joint tissue to respond to IL-23 by secreting proinflammatory cytokines. The IL-23-mediated enthesitis is reduced in the presence of IL-17 and IL-22 neutralizing antibodies; however, in contrast to IL-22, IL-17 alone is not sufficient to induce enthesitis [135].

## 3. IL-17 and Inflammatory Rheumatisms

### 3.1. IL-17 in Autoimmune Diseases

#### 3.1.1. Rheumatoid Arthritis

RA is the most frequent autoimmune arthritis in the world affecting around 1% of general population. It is a public health issue because of its chronicity and the progressive joint destruction experienced by some patients. The disease is characterized by inflammation of the synovium with a T cell, B cell, and proinflammatory cytokine infiltration. Etiology and pathophysiology of RA are not fully understood but an immunological conflict may precede the development of clinical stages of the disease [136]. Various environmental factors influence the development of the disease on specific genetic basis. These external and internal factors are not completely known, but are subjects of intense research. Complex immune modulator interactions resulting from this immunological conflict are at play in the joint, and therapeutic strategy largely uses this pathogenesis concept. A great range of immunomodulatory molecules is available in RA treatment from steroids to biologic and nonbiologic disease modifying antirheumatic drugs, of which the most used are, respectively, TNF-*α* blockers and methotrexate. Among proinflammatory cytokines, IL-17 axis seems to be of importance in RA pathophysiology, and both Th17 cells and mast cells have been described as IL-17 sources in inflamed joints of RA patients [102, 103]. Synoviocytes have been shown to produce CCL20 in autoimmune arthritis like RA, thus recruiting Th17 cells via CCR6 [103].

Autoimmune arthritis in animal models has been long considered as Th1 dependent. However, accumulating evidence is now in favor of a crucial role of Th17 cells. In CIA, development of joint destruction remains present in IFN-*γ* receptor-deficient mice [137, 138], whereas disease activity is markedly reduced in IL-17-deficient mice [82] as well as after blockade of IL-17 [97]. In a quite different model, RAG-deficient mice receiving naive CD4^+^ T cells from SKG mice, that are genetically prone to spontaneously develop chronic autoimmune arthritis, exhibit a Th17-dependent polyarthritis [139]. It has been shown that IL-1-receptor-antagonist- (IL-1Ra-) deficient mice develop spontaneous arthritis secondary to their increased sensitivity to IL-1 [140], and T cells play a critical role in this model since IL-1Ra deficient mice lacking T cells do not develop arthritis [141]. Interestingly, IL-1Ra-deficient mice present increased number of Th17 cells, and spontaneous development of arthritis is abrogated when associated with IL-17 deficiency (or IL-17 neutralization) [140, 142], demonstrating the great involvement of Th17 cells in this IL-1-driven arthritis model. However, after the onset of arthritis, neutralization of IL-17 prevents any further increase of the disease but does not reduce the arthritis score [140]. A critical role of IL-17 in development of arthritis has also been observed in F759 mice (characterized by increased STAT3 activation) [143] and specific-pathogen-free K/BxN mice treated with neutralizing anti-IL-17 antibody [144], two models of mice predisposed to develop T cell-dependent arthritis. All these results are in favor of a major role of Th17 cells in the development of T cell-dependent arthritis in mice.

Further experiments have explored the role of Th17 cells in autoimmune arthritis. On one hand, Th17 cells are proinflammatory; they are responsible for inducing the migration of innate immune cells with, as a result, an increase in the production of proinflammatory cytokines, chemokines, and matrix-degrading enzymes from these cells [145]. In addition, circulating Th17 cells from RA patients have the propensity to induce IL-6, IL-8, and MMP expression by RA synoviocytes [104], further pointing out the pathogenic role of Th17 cells in joint inflammation and degradation. On the other hand, Th17 cells promote autoimmunity; they generate the production of autoantibodies in several mouse models by enhancing germinal center formation, for example [146].

Beside Th17 cells, *γδ* T cells also contribute to IL-17 production in inflamed joints in the CIA mouse model [52, 147]. Although these IL-17 innate producers exacerbate CIA in mice [52], a recent study by Pöllinger et al. showed that in this mouse model of arthritis, Th17 cells, rather than IL-17^+^ *γδ* T cells, drive osteoclast-mediated joint degradation [147].

In human RA, there is some evidence of IL-17 involvement. Metawi et al. determined that IL-17 serum levels are higher in patients with inflammatory arthritis compared to healthy controls [148]. Th17 and Th22 cells have been found increased in peripheral blood of patients with RA, and levels are positively correlated with disease activity [149, 150]. IL-17 has also been found in joint tissue in higher quantity in RA than in osteoarthritis. It was linked in the same study to the production of matrix degradation molecules further proving the role of IL-17 in the pathophysiology of the disease [151]. Results on *γδ* T cells in mice are in line with human data showing that *γδ* T cells are not a prominent source of IL-17 in patients with RA [152].

Taken together, the IL-17/Th17 pathway seems greatly involved in the initiation process of autoimmune arthritis as well as in the inflammation stage of the disease, and IL-17-producing cells represent an attractive target in RA treatment.

#### 3.1.2. Systemic Lupus Erythematous

SLE is an autoimmune disease characterized by its chronicity and the reciprocation of flare and remission periods. It can affect a lot of organs, among them joints, kidneys, skin, or nervous system [153]. Patients with SLE usually exhibit antinuclear antibodies, whose pathogenicity remains unclear. The prevalence rate displays large worldwide variations, due to genetic and environmental factors, and SLE affects around 0.2 to 2 in 1000 individuals [154]. It is predominantly a disease of women with a mean sex ratio of 1 to 9, male to female. Level of disease severity is extensive, and treatments range from preventive measure and hydroxychloroquine to heavy immunosuppressive drugs.

Pathophysiology is far from fully understood but great advances have been made in the past few years. It involves complex interaction between environmental and genetic factors, and the presence of circulating autoantibodies directed against intracellular antigens, such as DNA, appears to be one of the major events in disease initiation [153]. These autoantibodies are involved in the pathogenesis since they complex with antigens, thus activating effector responses. The resulting tissue destruction exposes more intracellular antigens and sustains the reaction. Throughout the importance of these autoantibodies, SLE has traditionally been considered as a B cell-dependent disease. However, there is increasing evidence that T cells have a major place in SLE mechanisms. In this context, the role of Th17 cells during SLE has recently become subject of increasing attention [155].

It has been shown that the phenomenon of organ injury following ischemia is greatly dependent on Th17 cells in MRL/lpr mice, a lupus-prone model of mice [92, 156]. This is partly reversed by CD4 depletion or IL-17 deficiency, especially regarding tissue damages. At baseline, MRL/lpr mice present a higher frequency of IL-17-producing cells than nonautoimmune mice like B6 strain (unpublished data, [155]). Higher production of IL-17 is observed from SNF1 (lupus-prone mice) splenocytes cultured with nucleosomes than from B6 splenocytes [157]. A decrease of both IL-17 production and of Th17 cell infiltration in the kidney is found altogether with clinical improvement observed after tolerance induction with a histone-derived peptide. Taken together, these results confirm that Th17 cells are increased in lupus models, but they also seem to be involved in pathogenicity.

Similar IL-17/Th17 involvement is demonstrated in BXD2 mice (a strain of mice genetically engineered to develop autoimmune manifestations), where the humoral response is strongly increased. This is not independent of the presence of IL-17-producing cells since they have been demonstrated to have a major impact on germinal center development in the spleen [158]. Again, besides their proinflammatory profile, Th17 cells play a direct role in autoimmunity generation. In this context, the recent description of T follicular helper (TFH) cells, T cells helping B cells in an extrafollicular location, is of great importance [159]. This cell population has been observed in lupus-prone strains of mice. Those cells produce IL-17 and IL-21, the latter playing the job of helping B cells. However, it has been proposed that high production of IL-17 favors IL-21 secretion, giving an indirect role to IL-17 in the generation of autoimmunity by TFH.

In human, IL-17 is able to increase immunoglobulin production and thus anti-DNA antibodies in cells from SLE patients [155]. Indirect evidence of the role of IL-17 in human SLE is the increased level of that cytokine along with IL-23 and IL-21 in patient sera [160–162]. The origin of IL-17 seems to be CD4^+^ T cells and CD3^+^ double negative T cells. Patients with SLE have increased number of circulating IL-17-producing Th17 cells and CD3^+^ CD4^−^ CD8^−^ T cells than healthy controls, and the frequency of Th17 cells correlates with disease activity [98, 162–164]. More interestingly in the pathogenesis point of view, IL-17 has been histologically found in lupus nephritis, and IL-17 expression positively correlates with disease activity [160, 164, 165]. Finally, genetic associations with SLE have been highlighted with Th17-associated molecule polymorphisms as IL-21, and genetic variants decreasing Th17 differentiation are associated with a higher risk of developing SLE [166]. The very recent identification of IL-17-producing B cells as important players to combat trypanosome infection [48] begs the question of whether IL-17 production by B cells in SLE is dysregulated.

#### 3.1.3. Systemic Sclerosis

SSc is a rare connective tissue disease characterized by excessive extracellular matrix deposition in internal organs, like skin and lungs [167]. It affects approximately 1 per 10000 adult individuals and is highly dependent on the geographical location [168]. The ratio of women to men is about 4 : 1. Although survival in SSc has improved over the past several decades, SSc is still associated with a poor outcome. Despite the heterogeneity of the disease, disfigurement associated to cutaneous lesions, arthritis, fatigue, and dyspnea recapitulate the majority of patient complains. Besides, the major causes of invalidity and impairment of vital prognosis are digital ulcers, lung fibrosis, and pulmonary arterial hypertension. Therapeutic weapons used in SSc are largely nonspecific but lead to a slight decrease in mortality rates. Clinical progresses are mostly linked to vascular treatments and immunosuppressive strategy, although widely used in severe cases, such therapies have limited efficiency and are associated with significant side effects [169].

To date, SSc pathophysiology is still largely unknown, explaining the poor effectiveness of therapeutic strategy in SSc. Pathology includes vascular abnormalities, immune activation, and fibrosis [170], but the relationships between the three entities are still matter of debate. Accumulating evidence is in favor of a role of T cells in those mechanisms [167]. First, genetic studies indicate that most of the gene polymorphisms associated with SSc involve genes coding for molecules controlling T cell differentiation or activation, some shared with other autoimmune disorders like SLE [171, 172]. Second, histological examination of SSc skin during the early oedematous inflammatory phase of the disease demonstrates the presence of mononuclear cell infiltrates containing T cells, with perivascular distribution, preceding the development of fibrosis and overt vasculopathy [173]. These findings led to the hypothesis that T cells provide important stimuli that drive collagen synthesis in fibroblasts, propelling these cells to the forefront of SSc pathophysiology. Defining the T cell subsets at play has been the next challenge, and this issue is far from completely solved. On one hand, Th2 cells, mainly through their prototypic cytokines, are certainly involved in the disease fibrosis process [174]. On the other hand, some evidence points out the role of IFN-*γ* and Th1 cells [175]. However, accumulating reports over the last few years highlighted IL-17 and Th17 cells as important actors of the disease [176, 177].

IL-17 has been shown to be involved in the development of bleomycin-induced mouse lung fibrosis in an IL-1-dependent way [178]. In two different models of mouse SSc, importance of IL-17 was suggested. In bleomycin-induced skin fibrosis, the loss of IL-17 decreases the fibrotic process, and higher IL-17 mRNA levels are found than in wild-type skin [179]. IL-17 deficiency also attenuates skin thickness in tight skin 1 (TSK-1) mice, a strain of mice presenting spontaneous mutation in fibrillin-1 gene and used as a model of SSc. Furthermore, IL-17 stimulates directly collagen synthesis in rodent fibroblasts [180]. Animal models are poorly relevant for SSc human pathogenesis, but these are first clues of IL-17 involvement.

Increased levels of IL-17 are detected in the sera and bronchoalveolar lavage fluids of SSc individuals [181]. We and others observed an increase in Th17 and Th22 cells frequency in peripheral blood of SSc patients, further enhanced by some SSc treatment via monocyte production of IL-23 among others [176, 182]. In the skin of SSc patients, we recently showed an increase in IL-17-producing cells with an inverse correlation to the skin fibrosis score [177]. *In vitro*, IL-17 is able to partially inhibit the expression of *α*-smooth muscle actin induced by TGF-*β* and to induce the secretion of MMP1 in human dermal fibroblasts, and conversely to rodent, human fibroblasts do not produce collagen in response to IL-17. The difference in mouse and human responses to IL-17 may be explained by species-specific characteristics in the IL-17 biology, as it has been previously seen for Th17 differentiation, bringing caution towards murine models regarding the extrapolation of therapeutic strategies [183]. The hypothesis that, in humans, IL-17 and Th17 cells in SSc could be more related to inflammation, autoimmunity, and possibly to the generation of autoantibodies is seductive, and until now, no direct argument for the role of this pathway in the SSc arthritis pathophysiology has been reported in the literature.

### 3.2. IL-17 in Inflammatory Arthritis

#### 3.2.1. Psoriatic Arthritis

Psoriatic arthritis (PsA) belongs to the spondylarthritis group of diseases and is characterized by a chronic inflammation of joints and skin. Peripheral and axial joints can be affected by the disease, with a potential breach of enthesis and synovial membranes in the meantime. PsA is a frequent inflammatory rheumatism, nearly as frequent as RA, as it concerns about 0.3 to 1% of general population [184]. Its presentation and course are highly variable. Mostly, skin involvement precedes joint inflammation, but the osteoarticular lesions may be present before the development of psoriasis in 10% of cases. Psoriasis is completed with arthritis in one-third of the patients during the development of the disease. The persistence of inflammation in joints can lead to destruction and severe disabilities. Until now, therapeutic strategies in PsA are often directly inspired by those used in RA.

Nevertheless, despite common features, PsA differs from RA in some aspects. The early events in PsA pathogenesis occur in genetically predisposed subjects and are mediated by T cells interaction with antigen-presenting cells. The location of the first immune conflict is not really defined but TNF-*α* seems to play an important role, and TNF-*α* blockers remain highly used and efficient treatments of the disease. The first antigen is still unknown; nevertheless, it induces a T cell-specific reaction followed by a proinflammatory cytokine secretion cascade. PsA, as with other rheumatic inflammatory disorders, was considered until recently as a Th1-dependent disease with IFN-*γ* playing an important role in the generation of that cascade. Psoriatic disease encompasses psoriasis and the involvement of musculoskeletal and gastrointestinal and ocular systems. Thus, PsA pathogenesis is closely connected to that of psoriasis. Much less information is available in PsA pathogenesis than in skin psoriasis, but both have susceptibility associated with alleles of the *IL-12B *and *IL-23R *genes [185, 186]. Moreover, IL-12/23 p40 subunit is elevated in sera of PsA patients [187]. Given the importance of IL-23 in Th17 biology, it suggests a role of this subset in PsA. Finally, with the importance of the IL-17 pathway in psoriasis and in autoimmune arthritis murine models, the role of IL-17/Th17 has been naturally evoked in PsA. IL-17 is increased in PsA synovial tissue and fluid. IL-17RA is overexpressed by PsA synoviocytes [188], is functionally active, and regulates the synoviocyte secretion of proinflammatory cytokines and matrix metalloproteinases tightly involved in the joint damages observed in PsA. Another indirect evidence of the role of IL-17 in PsA pathogenesis has been recently reported with the involvement of the adaptor protein Act1 in the disease through genome-wide association or functional studies [189, 190]. The correlation between disease activity and levels of IL-17 or Th17 cytokines in synovial fluid is variable [188, 191]. Differences in disease patterns or treatment regimen in studied population could be the reason of this discrepancy. It appears that the earlier and the more free of any treatment the patient is, the more correlated is the disease activity with Il-17 rates. These findings are in accordance with the bone erosive role of IL-17 demonstrated *in vitro* and highlight its role not only in the skin, but also in all major components of psoriatic disease. Whether or not innate immune cells contribute to IL-17 production in PsA is still unknown.

#### 3.2.2. Ankylosing Spondylitis

AS is a systemic disease characterized by enthesopathy and ossification of the joints [192]. It is the most frequent member of the spondylarthritis group, and its prevalence ranges between 0.2 and 1.2% of general population. Affecting more often men than women (3 : 1), its peak is around the third decade of life. The clinical characteristic of AS is the axial joint damages, most notably the sacroiliac joints. Ossification and ankylosis are typical of the disease, but wide extraarticular manifestations can occur such as digestive and ocular but also and less frequently heart or lung damages. These extraarticular manifestations, expressing the systemic nature of the disease, are also involved in the therapeutic strategy. All spondyloarthropathies are associated with human leukocyte antigen (HLA)-B27 expression, the highest association is yet in the case of AS, as 90% of patients are HLA-B27 positive.

The pathogenic role of HLA-B27 in AS has been and is still debated. Hypotheses are as varied as arthritogenic peptide presentation, aberrant folding of surface heavy chains, or enhancing of intracellular microbial survival [193]. AS is an inflammatory disease, and to date, no specific antibodies have been identified. The immune system is still highly involved in the disease pathogenesis. The privileged association with HLA-B27 might be a clue for CD8^+^ T cells involvement. Even so, transgenic HLA-B27 rats, used as prototypic AS rodent model, present colitis and arthritis independently of CD8^+^ T cells, raising the question of other important players in the disease pathogenesis. Some proinflammatory cytokines are constantly increased in AS patients, such as TNF-*α*, IL-6, and IL-2 receptors [194].

Pathophysiology of AS has been recently enriched with genetic and more precisely genome-wide association studies, linking the disease to *IL-23 receptor* gene [195]. Other findings are in favor of the IL-23/IL-17 pathway involvement notably in murine models [36].

SKG mice are genetically prone to develop autoimmune arthritis, and curdlan injection can drive spondylarthritis symptoms [196]. The pathology is at least partly driven by IL-17-secreting *γδ* T cells and IL-17 deficiency ameliorates symptoms [197]. IL-23 was also very recently shown to be directly involved in AS and more precisely in the development of enthesitis in a collagen antibody-induced arthritis mouse model [135]. IL-23 induces AS in mice through the activation of IL-17/IL-22-producing ROR*γ*t^+^CD3^+^CD4^−^CD8^−^ T cells directly located in the entheses. The mice phenotype is not only enthesitis, but it also recapitulates all features of AS [135].

In human, IL-17 is expressed in sacroiliac joints biopsies from AS patients. However, many arguments converge towards the involvement of innate immune cells. Notably, mast cells, neutrophils, and *γδ* T cells seem to be good candidates as IL-17-secreting cells in AS joints [101]. More indirectly, IL-23R expression has been found increased in active sites of the disease in AS patients, such as tendon-bone junction and aortic root [135]. Finally, Bowness et al. recently raised an interesting issue by establishing a link between HLA-B27 and IL-17 pathway [198]. They showed that APC expressing B27 *β*2 microglobulin-free heavy chain homodimers are prone to induce the proliferation of specific Th17 cells. These cells produce IL-17 and/or IFN-*γ* due to a high plasticity and are found in AS patients, suggesting their involvement in the disease pathogenesis [198].

## 4. Therapeutic Applications: Strategies and Molecules Targeting the IL-17/Th17 Pathway in Inflammatory Rheumatisms

Regarding all the findings involving the IL-17/Th17 pathway in inflammatory rheumatisms, the idea to target this pathway has become more and more attractive. The topic is discussed as a strategy more than a single treatment because of the complexity of the inflammatory process at play in IL-17 biology. There are several potential targets in the cascade leading to IL-17 effects, and we will describe those currently under consideration.

Standard treatment in RA is association of methotrexate together with a TNF-*α* blocker when an adequate response is not achieved.

### 4.1. Direct Targeting of IL-17

Directly targeting the cytokine is a classic strategy in biologic development based on monoclonal antibodies production. Owing to TNF-*α* blockers such as monoclonal antibodies (infliximab, Remicade and adalimumab, Humira) or receptor-targeting fusion protein (etanercept, Enbrel), a great experience and hindsight regarding this type of treatment exist in rheumatology.

Several molecules are in the pipeline of development with some advanced data of clinical trials (http://www.biocentury.com/targets/il-17).

Two monoclonal antibodies directed against IL-17 are currently tested in humans. Ixekizumab (LY2439821), a humanized hinge-modified IgG4 IL-17-specific antibody developed by Lilly is in a phase III trial for psoriasis and for PsA (clinicaltrial.gov, identifiers NCT01597245, NCT01624233, and NCT01646177). It has already completed a phase I and a phase II trial in RA ([199] and clinicaltrial.gov, identifiers NCT00966875). Secukinumab (AIN457), a fully human IL-17-specific IgG1k monoclonal antibody generated by Novartis is also in advanced clinical development. This molecule is in phase III for chronic plaque psoriasis, PsA, RA, and AS, and in phase II for chronic noninfectious uveitis [200, 201].

First results from trials with both antibodies are quite encouraging with an improvement in symptoms and a good safety profile. In a double-blind, placebo-controlled, parallel-group, phase IIA study (*N* = 36) in moderate to severe psoriasis, a single infusion of secukinumab (3 mg/kg) resulted in rapid and sustained improvement of psoriasis symptoms, with at week 4, 83% of secukinumab patients versus 11% of placebo patients achieving significant efficacy [201]. The effect was less impressive in a double-blind, placebo-controlled phase IIA study assessing safety and efficacy of subcutaneous secukinumab in PsA. The difference in rate of American College of Rheumatology 20 score (ACR20) response at week 6, with secukinumab or with placebo, was not statistically significant. Significant efficacy compared to placebo was finally reached at weeks 12 and 28.

Other IL-17-targeted antibodies are in early clinical development, such as SCH-900117 and RG4934. Brodalumab (AMG827), a human anti-IL-17RA antibody developed by Amgen/MedImmune has shown remarkable efficacy for the treatment of psoriasis in a phase II double-blind, placebo-controlled, dose-ranging study [202] and is currently in Phase II trial in RA and PsA [203].

In addition, new strategies under consideration aim to inhibit biological activities of more than one cytokine. For example, Roche is testing a blocking antibody targeting both IL-17A and IL-17F (RG7624, no clinical information available; Roche website and [36]). In addition, based on the synergistic activities of IL-17 and TNF-*α*, an anti-TNF-*α*/IL-17 bispecific neutralizing antibody (ABT-122, Abbott) is being tested in phase I in RA (Abbott website and [36]).

### 4.2. Indirect Targeting of the IL-17 Pathway

Current or in development tools for targeting the IL-17/Th17 pathway are also showing promising results to treat inflammatory rheumatisms.

#### 4.2.1. IL-23 and/or IL-12 Targeting

Ustekinumab (Stelara) is a human monoclonal antibody engineered by Janssen Biotech that targets the p40 subunit of IL-12 and IL-23, and therefore inhibits both IL-23 and IL-12 signaling. It has been approved for psoriasis treatment since 2009 [204]. In a phase II double-blind, placebo-controlled, crossover study in patients with active PsA, subcutaneous injections of ustekinumab significantly reduced signs and symptoms of PsA and improved skin lesions and physical function in patients [205, 206]. Ongoing phase III trials will establish the benefice/risk profile of ustekinumab in this disease.

Given the major role of the IL-23/IL-17 pathway in inflammation and autoimmunity, new drugs in development specifically aim to neutralize only the IL-23 pathway and three anti-p19 neutralizing antibodies, MK-3222, CNTO 1959, and AMG 139, respectively, developed by MERCK, Janssen Biotech, and Amgen/MedImmune are currently in clinical trials for psoriasis, as well as Crohn's disease for AMG 139 [203, 207]. Only available clinical data reported that administration of MK-322 in psoriatic patients markedly decreases cutaneous inflammation. Such effects are associated with a significant reduction of T cells, dendritic cells, neutrophils, and macrophages in the inflammatory infiltrate [208]. These promising results suggest that neutralization of IL-23 but not IL-12 could be sufficient to inhibit downstream signaling cascades involved in disease development.

In this context, clinical trials assessing the effect of IL-23 blockade could also represent an interesting approach for the treatment of inflammatory rheumatisms.

#### 4.2.2. IL-6 Targeting

IL-6 is another proinflammatory cytokine involved in the development of joint inflammation, and Tocilizumab (RoActemra) has proven its efficacy for few years in RA patients and is also approved since 2011 for treatment of systemic juvenile idiopathic arthritis [209]. It is a humanized anti-IL-6 receptor monoclonal antibody developed by Roche that binds both soluble and membrane bound IL-6 receptor and prevents IL-6 binding to its receptors [210]. The use of Tocilizumab is growing in RA patients, particularly in those with an inadequate response to methotrexate or TNF-*α* inhibitors therapies [211–213]. It is administered through intravenous infusion every four weeks and can be used as a monotherapy or in combination with methotrexate. Interestingly, a recent clinical trial compared the efficacy of tocilizumab versus adalimumab (anti-TNF-*α* antibody) as monotherapy in RA and revealed a significant greater disease improvement in patients treated with tolicizumab [214].

While IL-6 targeting is showing efficiency in RA, tolicizumab therapy has been disappointing in SpA patients [214, 215].

Other IL-6 targeting therapies in development include BMS945429, a humanized anti-IL-6 antibody engineered by Alder Biopharmaceuticals, and sarilumab, a human anti-IL6 receptor *α* (IL-6R*α*) antibody codeveloped by Regeneron and Sanofi [214]. Phase II, double-blind, randomized, placebo-controlled studies showed that BMS945429 or sarilumab treatment associated with metothrexate induced significant improvement of disease activity in patients with active RA and inadequate response to methotrexate [214, 216]. If sarilumab is giving promising results for RA treatment, a phase II clinical trial assessing the effect of this anti-IL-6R*α* antibody in AS did not show any significant efficacy [214].

#### 4.2.3. IL-1 Targeting

IL-1 targeting started in 1993 with anakinra (Kineret), a recombinant IL-1 receptor antagonist developed by Amgen, which inhibits both IL-1*α* and IL-1*β* activities. It was approved for RA treatment in 2001 and is being tested in a number of diseases, including autoimmune and autoinflammatory diseases (e.g., severe atopic dermatitis, osteoarthritis of the knee), but also in diseases that are not inflammatory, like heart failure and type 2 diabetes [217]. Treatment with anakinra is fastidious for RA patients as it has to be daily injected because of its short half-life, and its efficacy is similar to other biologics; therefore, anakinra is not among first-line therapies. 

Two other IL-1 targeting agents have been approved for the treatment of cryopyrin-associated periodic syndromes: rilonacept (Arcalyst, Regeneron), a soluble decoy receptor, and canakinumab (Ilaris, Novartis), a humanized monoclonal antibody against IL-1*β*. Several additional agents blocking IL-1 are in clinical trials in several diseases (e.g., stroke, diabetes, and chronic inflammatory diseases) and target the IL-1 receptor, IL-1*α*, IL-1*β*, or caspase 1 (crucial for IL-1*β* activity). IL-1*β* and IL-1 receptor neutralizing antibodies are currently tested in arthritis and joint diseases [217].

### 4.3. Targeting Signaling Pathway Molecules

Transcription factors modulators are very trendy among developing therapeutic strategies, and in USA, it represents 13% of current U.S. Food and Drug Administration-approved drugs.

#### 4.3.1. ROR*γ*t and/or ROR*α* Inhibition

As mentioned before, ROR*γ*t is both necessary and sufficient for induction of IL-17 production in human cells. This transcription factor represents potential therapeutic target and can be blocked by specific inhibitors. As ROR family is earlier in the IL-17 production cascade, its targeting can not only diminish the secretion of this cytokine but also favors the shift from Th17 towards regulatory T cells. This more complex effect could be of interest in the treatment of autoimmune diseases where an imbalance between effector and regulatory T cells has been observed. Nevertheless, as not being the only transcription factor involved in Th17 differentiation, ROR*γ*t inhibition might not have a complete IL-17 suppressive effect.

Small molecules have been described as capable of inhibiting the ROR family and thus target the IL-17 pathway. Digoxin, a cardiac glycoside, has been shown to be a specific inhibitor of ROR*γ*t independently of ROR*α* [218]. SR1001 is a high-affinity synthetic ligand for both ROR*α* and ROR*γ*t, and Solt et al. demonstrated its inhibitory effect on differentiation and function on Th17 cells. It induces conformational changes in the molecule, leading to a higher affinity for corepressors and lower for coactivators [219]. Ursolic acid is a third molecule involved in the inhibition of the IL-17 pathway through its action on the ROR family member ROR*γ*t [220]. Evidence for this molecule efficacy on IL-17 modulation in autoimmune diseases is still limited to *in vitro* or murine studies. To our knowledge, no molecules directly targeting ROR family are currently at clinical trial phase in human. Nevertheless, because it regulates ROR*γ*t expression, forkhead box P3 (FoxP3) upregulation can indirectly induce a decrease of IL-17 production. Molecules such as simvastatin (3-hydroxy-3-methylglutaryl coenzyme A reductase inhibitor), commonly administrated in atherosclerosis treatment, enhance FoxP3 expression and inhibit the production of IL-17 [221].

#### 4.3.2. Janus Kinases (JAK)-STAT3 Inhibition

As stated before, IL-23, IL-6, and IL-21 are involved in Th17 differentiation, and they all act through the JAK-STAT signaling pathway. Thus, STAT3 has become an interesting potential target, at the convergence point of different upstream activators. It has been recently shown *in vitro* that STAT3 inhibition in synovial T cells of RA patients suppresses Th17 pathway [222]. Research in that field remains at the discovery stage; this demonstration is being done using siRNA, and no therapeutic molecules are currently tested in human clinical trials in the domain of autoimmune diseases. However, clinical trials are completed in the area of oncology, and this experience might be useful in a near future in the immunology field as it happened for rituximab [223].

On the other hand, the anti-JAK strategy is far much advanced with the development of tofacitinib, an oral JAK inhibitor, tested in a phase III clinical trial in the treatment of RA [224]. This molecule directly inhibits the production of IL-17 and IFN-*γ*, resulting in a decrease of proinflammatory cytokine production and synovitis [225]. Tofacitinib effect is also assessed for other inflammatory diseases involving IL-17 such as psoriasis or ankylosing spondylitis. STAT3 and JAK are closely linked as the second phosphorylates the first leading to its nuclear translocation and biological activity [226]. Inhibition of JAK molecules might have several effects, but it was demonstrated that tofacitinib inhibits IL-17 secretion *in vitro* [227]. This could be one of its mechanisms of action.

#### 4.3.3. Phosphoinositide 3-Kinase *δ*-Subunit (PI3K*δ*) Inhibition

The PI3K/Akt pathway is involved in both the pathogenesis of RA and IL-17 production [228, 229]. It is therefore a potential target in the scope of research in the autoimmunity therapeutic agent field. ZSTK474, a general PI3K inhibitor, has shown the ability to inhibit synovial inflammation, osteoclastic activity, and finally collagen-induced arthritis *in vitro* and in murine models [230]. Class 1 PI3K exhibits two isoforms of the catalytic subunits, p110*γ* and p110*δ* that are enriched in leucocytes. The selective inhibition of one of those subunits has attracted a major interest due to data of *in vitro* models notably in collagen- or in antigen-induced arthritis [231–233]. Finally, the rationale seems to be strong enough in RA and SLE literature to bridge the gap between the bench and bedside research. Promising results have been obtained with the p110*δ* inhibitor CAL-101 in the field of lymphomas giving reassuring data about the safety of this molecule, but clinical trials are still ongoing, and data remain unpublished [234].

## 5. Conclusion

It is now clearly demonstrated that IL-17 is deeply involved in autoimmune and inflammatory processes. Joint is a prime target of IL-17 action, and all compartments appear to be concerned by the action of this cytokine. Mechanisms are intensively studied because of potential therapeutic strategies that may arise. All of the pathogenesis is far from elucidated, but despite this fact, many molecules that target the IL-17/Th17 pathway are already under development or even tested in clinical trials for the treatment of autoimmune or inflammatory diseases.

Very recently, a new mechanism has been pointed out in the IL-17-autoimmunity interaction, bringing a new component in the system going awry: salt. Two different studies showed that in an isotonic culture medium, an elevated sodium chloride (NaCl) concentration promotes the differentiation of Th17 cells *in vitro* [235, 236]. One even brought *in vivo* evidence of the proautoimmune effects of a high-salted diet. These intriguing studies reassert the role of environmental factors in these diseases. It also emphasizes that there is not one Th17 cell but Th17 cells. These cells act differently to protect or to damage tissues, leading to an even more complicated story than it was initially imagined, and certainly quite far from what is observed in animal models.
